# The mycobiome of root canal infections is correlated to the bacteriome

**DOI:** 10.1007/s00784-016-1980-3

**Published:** 2016-10-22

**Authors:** Ilona F. Persoon, Mark J. Buijs, Ahmet R. Özok, Wim Crielaard, Bastiaan P. Krom, Egija Zaura, Bernd W. Brandt

**Affiliations:** 1 Department of Preventive Dentistry, Academic Centre for Dentistry Amsterdam (ACTA), University of Amsterdam and Vrije Universiteit Amsterdam, Gustav Mahlerlaan 3004, 1081 LA Amsterdam, The Netherlands; 2 Department of Endodontology, Academic Centre for Dentistry Amsterdam (ACTA), University of Amsterdam and Vrije Universiteit Amsterdam, Gustav Mahlerlaan 3004, 1081 LA Amsterdam, The Netherlands

**Keywords:** Bacteria, Fungi, Microbiome, Next-generation sequencing, Primary endodontic infection

## Abstract

**Objectives:**

Bacterial infection of the root canal system causes apical periodontitis. Less is known about the role of fungi in these infections. This study aimed to assess the fungal prevalence, abundance, and diversity of root canal infections, as well as the relation between fungi and bacteria present in different parts of the root canal.

**Materials and methods:**

Twenty-six teeth with primary apical periodontitis were extracted, split in apical and coronal root segments, and cryo-pulverized. Bacteriome profiles of 23 teeth were analyzed based on the V3-V4 hypervariable region of the 16S ribosomal RNA gene. Mycobiome profiles of six teeth were analyzed based on the internal transcribed spacer (ITS) 1 or ITS2 region. Samples were sequenced on the Illumina MiSeq platform.

**Results:**

A total of 338 bacterial operational taxonomic units (OTUs), 28 ITS1 OTUs, and 24 ITS2 OTUs were identified. *Candida* and *Malassezia* were the most frequently identified fungi. No differences could be found between the bacteriome and mycobiome profiles of the apical and coronal root segments. The bacteriome of fungi-positive root segments contained more *Actinomyces*, *Bifidobacterium*, four different *Lactobacillus* OTUs, *Propionibacterium*, and *Streptococcus*. A Spearman correlation matrix between bacteriomes and mycobiomes identified no correlations, but separate clusters could be observed.

**Conclusions:**

A considerable proportion of the root canal infections contain fungi, although fungal diversity is limited. However, when fungi are present, the composition of the bacteriome is clearly different.

**Clinical relevance:**

Interaction between bacteria and fungi in root canal infections may complicate the infection and require alternative treatment strategies.

**Electronic supplementary material:**

The online version of this article (doi:10.1007/s00784-016-1980-3) contains supplementary material, which is available to authorized users.

## Introduction

Microbial infection of the dental root canal system causes the host to respond with an inflammatory process at the root apex, called apical periodontitis [1]. Root canal treatment aims at inactivation and removal of the microorganisms and their toxic metabolites, in order for the inflammation to resolve [2]. However, the complex root canal system [3] and microorganisms residing in resilient biofilm communities [4] impede sufficient cleaning, which can lead to persistent apical periodontitis [5].

Traditional methods to study the microbiology of root canal infections range from cultivation to polymerase chain reaction (PCR), cloning, and Sanger sequencing [6]. Recently, next-generation sequencing techniques have shown that root canal infections are far more diverse than previously considered [7]. This technique allows an open-ended analysis with an increased sampling depth, which facilitates identification of many more species including less abundant species. Between 339 and 2168 different species-level operational taxonomic units (OTUs) of bacteria have been identified in the bacterial community, the bacteriome, of root canal infections, and 13–98 OTUs were found per root canal [7–14]. This immense diversity drives a dynamic community that capacitates the bacteria to sustain periapical inflammation and persist even after treatment.

Next to bacteria, fungi can also be involved in root canal infections and have been identified in 0.5–55 % of the infections [15]. These studies have primarily identified *Candida albicans* using cultivation, microscopy or PCR. Next-generation sequencing of DNA from oral rinse and saliva samples has shown that fungi are highly prevalent and that the fungal community, the mycobiome, is diverse [16, 17]. It has not previously been reported whether root canal infections also have a complex mycobiome. Fungi can function independently of, but often interact with, bacteria. This can increase virulence of either or both types of microorganisms [18, 19], which may enable microbial persistence and result in a treatment-resistant microbial community. The contribution of fungi to the complex ecology of root canal infections has not been studied yet, but is highly relevant as it may give leads to improve current root canal treatment.

Thus far, microbiological research of root canal infections has mostly applied traditional sampling methods such as paper point sampling. This method can only sample the main root canals and cannot sample the complete root canal system [20]. Moreover, these samples do not permit differentiation between the apical and coronal niche of the root canal [11]. Pulverization of extracted teeth permits analysis of the microorganisms in all the extremities of the root canal system, which can be separated for the apical and coronal halve of the root canal system [9, 11, 20]. Thus, paper point sampling and identification using cultivation or targeted closed-ended DNA methods limit the possibilities to fully uncover the microbial community responsible for apical periodontitis. Analysis of all microorganisms in the complete root canal system can be achieved using next-generation sequencing on pulverized root segments.

The aim of the present study was to assess the fungal prevalence, abundance, and diversity within primary root canal infections analyzed using next-generation sequencing on pulverized roots, as well as to assess the relation between fungi and bacteria present in two different parts of the root canal.

## Materials and methods

### Patient selection

This study was conducted in accordance with the ethical principles of the 64th WMA Declaration of Helsinki. The ethical committee of the Academic Centre for Dentistry Amsterdam approved the study (19 February 2009). Patients presented with teeth with apical periodontitis, and refrained from endodontic treatment, chose to have the tooth extracted. All participants gave informed consent that the teeth would be used for this study. Patients were included if they had premolars or molars that were asymptomatic and when they had apical periodontitis confirmed by a periapical radiograph. Patients were excluded if there was visible exposure of the pulp chamber or advanced periodontal disease.

### Sample processing and DNA extraction

All subsequent procedures were performed under strict aseptic conditions. After extraction, the tooth surface was vigorously scrubbed with gauzes soaked in 0.5 % sodium hypochlorite. This was inactivated by wiping off the tooth with a gauze soaked in 5 % sodium thiosulfate. The tooth was decoronated using a sterilized diamond disk under saline cooling, after which the root was split into an apical and a coronal segment with another sterile diamond disk, again under saline cooling. The segments were stored at −80 °C until further processing. Root segments were cryo-pulverized under aseptic conditions using a freezer mill (Spex Certiprep, Metuchen, NJ, USA) and stored at −80 °C in 5 mL RNA stabilization solution (RNA*later*, Qiagen, Hilden, Germany).

The powdered root segments were thawed and centrifuged for 5 min at 4500×*g*. The supernatant was removed, and the powdered root segment was resuspended in 200 μL lysis buffer (Mag Mini DNA Isolation Kit, LGC, Hoddesdon, UK) and transferred to 2-mL screw capped tubes (Sarstedt, Nümbrecht, Germany) with 350 μL zirconium beads (0.1 mm; BioSpec products, Bartlesville, OK, USA), 350 μL phenol saturated with Tris-HCl (pH 8.0; Carl Roth, Germany), and 120 μL 0.5 M ethylenediaminetetraacetic acid (EDTA) to reverse the binding of DNA to dentin [21]. Samples were placed in a Mini-BeadBeater-96 (BioSpec products, Bartlesville, OK, USA) for 4 × 2 min at 2100 oscillations/min. DNA was extracted using the Mag Mini DNA Isolation Kit.

A quantitative polymerase chain reaction (qPCR) was used to determine the amount of bacterial and fungal DNA. Primers specific to the bacterial 16S rRNA gene (forward: TCCTACGGGAGGCAGCAGT; reverse: GGACTACCAGGGTATCTAATCCTGTT; probe: 6FAM-CGTATTACCGCGGCTGCTGGCAC-BHQ1) [22] and the fungal 28S rRNA gene (forward: GCATATCAATAAGCGGAGGAAAAG; reverse: TTAGCTTTAGATGATTTACCACC; probe: CGGCGAGTGAAGCGGSAARAGCTC) were used [23].

To confirm aseptic processing of the teeth, qPCR using universal 16S and 28S rRNA primers was applied on two samples of extracted, sound teeth with vital pulps, processed similarly as the test samples. The 16S rDNA levels were below the detection limit and similar to the negative PCR controls, and therefore, the sample processing was considered adequate.

### Amplicon preparation

Bacterial amplicons of the V3-V4 hypervariable region of the 16S rRNA gene (341F forward primer: CCTACGGGNGGCWGCAG; [24]; 806R reverse primer: GGACTACHVGGGTWTCTAAT; [25]) were generated by PCR. These primers contained Illumina adapters and an 8-nucleotide index barcode sequence [26]. The amplification mix contained 2 U of Phusion HotStart II High fidelity polymerase (Thermo Scientific, Waltham, MA, USA), 1 U of Buffer Phusion HS II (5×), including 1.5 mM magnesium chloride (MgCl_2_; Thermo Scientific), 0.2 mM dNTP (Thermo Scientific), and 1 μM of each primer. To each reaction mix, 1 ng of DNA template was added. After denaturation (98 °C; 30 s), 33 cycles of denaturation (98 °C; 10 s), annealing (55 °C; 30 s), and extension (72 °C; 30 s) were performed, followed by an extension period (72 °C; 5 min). The amount of DNA per sample was quantified using the Quant-iT PicoGreen dsDNA Assay Kit (Invitrogen, Paisley, UK). The amplicons libraries were pooled in equimolar amounts and purified using the Illustra GFX PCR DNA and Gel Band Purification Kit (GE Healthcare, Eindhoven, The Netherlands). The quality and size of the amplicons were analyzed on the 2100 Bioanalyzer (Agilent Technologies, Santa Clara, CA, USA). Paired-end sequencing (2 × 301 cycles) of the DNA was conducted on the MiSeq platform (Illumina, San Diego, CA, USA) at the VU University Medical Center Cancer Center Amsterdam, Amsterdam, The Netherlands. The flow cell was loaded with 6 pmol DNA containing 50 % PhiX.

Fungal amplicons of the hypervariable internal transcribed spacer (ITS) regions ITS1 (ITS1F forward: CTTGGTCATTTAGAGGAAGTAA; ITS2 reverse GCTGCGTTCTTCATCGATGC) [27, 28] and ITS2 (ITS86F forward: GTGAATCATCGAATCTTTGAA; ITS4 reverse: TCCTCCGCTTATTGATATGC) [27, 29] were generated by PCR. All fungal primers contained adapter sequences to ligate to Nextera Set A Amplicon primers with 8-nucleotide index sequences (Illumina protocol, Part # 15044223 Rev. B). The amplification mix contained 2 U of Phusion HotStart II High fidelity polymerase, 1 U Buffer Phusion HS II (5×), including 1.5 mM MgCl_2_, 0.2 mM dNTP, and 1 μM of each primer. To each reaction, 1 ng of DNA template was added. After denaturation (98 °C; 30 s), 40 cycles of denaturation (98 °C; 10 s), annealing (55 °C; 30 s), and extension (72 °C; 30 s) were performed, followed by an extension period (72 °C; 5 min). PCR products were purified using solid-phase reversible immobilization (SPRI) beads [30]. The Nextera adapters were ligated using an 8-cycle PCR (Illumina protocol, Part # 15044223 Rev. B), and the amount of DNA per sample was quantified using the Quant-iT PicoGreen dsDNA Assay Kit. The amplicons libraries were pooled in equimolar amounts and purified with SPRI beads. The quality and size of the amplicons were analyzed on the 2100 Bioanalyzer (Agilent Technologies, Santa Clara, CA, USA). Paired-end sequencing (2 × 301 cycles) of the DNA was conducted on the MiSeq platform at the VU University Medical Center Cancer Center Amsterdam, Amsterdam, The Netherlands. The flow cell was loaded with 5.5 pmol DNA containing 50 % PhiX.

### 16S rDNA amplicon processing

Sequencing reads were merged [31], processed, and clustered using USEARCH version 8.0.1623 [32]. The minimum length of the merged read pairs was set at 380 bases and maximum length at 438 bases. Sequences were then quality filtered (max. expected error rate 0.002, no ambiguous bases allowed) and clustered into operational taxonomic units (OTUs) using the following settings: -uparse_maxdball 1500, only de novo chimera checking, and usearch_global with –maxaccepts 8 –maxrejects 64 –maxhits 1. The most abundant sequence of each OTU was selected using QIIME version 1.8.0 [33] and assigned a taxonomical classification using the RDP classifier [34] with a minimum confidence of 0.8 and the 97 % representative sequence set based on the SILVA rRNA database (release 119 for QIIME; [35]). In addition, the representative sequences were classified using the Human Oral Microbiome Database (HOMD) [36]. The HOMD-aligned sequence set (version 14.5; obtained from http://homd.org) was first trimmed to the V3-V4 region, after which it was converted to a nonredundant set of gap-free sequences to retrain the RDP classifier.

### ITS amplicon processing

The ITS sequence data was first filtered to remove reads with ambiguous bases, after which the reads were screened for the presence of the ITS primer, allowing one mismatch. Read pairs, containing the forward and reverse ITS primer one time, were then merged using USEARCH version 8.0.1623 [31] after removal of the primers, allowing “staggered” alignments and a length between 70 and 590 nucleotides. Next, the merged read pairs were quality filtered (max. expected error rate 0.002). Before clustering, the fungal ITS region was extracted from the sequences (to which the used ITS primers were reattached) using ITSx version 1.0.11 [37]. The ITS sequences were clustered similarly to the 16S rDNA data, after which the quality-filtered sequences were mapped to the OTU centroids. The most abundant sequence of each OTU was selected using QIIME version 1.8.0 [33] and assigned a taxonomical classification using the RDP classifier [34], with a minimum confidence of 0.8, and the UNITE database (QIIME release, version 7, dynamic use of clustering thresholds; file: sh_refs_qiime_ver7_dynamic_s_31.01.2016.fasta) [38].

### Statistical analysis

To correct for unequal sequencing depth, data was normalized by subsampling the 16S rDNA data at 6400 reads per sample, the ITS1 data at 6400 reads, and the ITS2 data at 4800 reads. Alpha-diversity (within-sample diversity) using the Shannon diversity index was compared using the Wilcoxon signed-rank test for comparing apical and coronal root fragments and the Mann-Whitney *U* test for comparing fungi positive and negative teeth.

OTU abundances were log2 transformed to normalize the distribution for principal component analyses (PCA). Compositional differences between groups were determined using one-way permutational multivariate analysis of variance (PERMANOVA) on the Bray-Curtis similarity indices of between-sample diversity. If significant, these differences were further analyzed by the linear discriminant analysis (LDA) effect size (LEfSe; version 1.0) method to identify the OTUs responsible for segregation into distinct groups [39]. The cutoff LDA value was kept at the default of 2.0. The level of significance was kept at the default of *α* = 0.05. Identified OTUs were tested for differences in abundance using the Mann-Whitney *U* test, adjusted for multiple testing using the false discovery rate (FDR) correction [40]. To test whether two root segment samples of a pair were compositionally more similar than root segments grouped by the presence of fungi or anatomical position, the Kruskal-Wallis test was performed on Bray-Curtis similarity indices. Post hoc analysis was done using the Mann-Whitney *U* test with an FDR correction, where the adjusted level of significance was q* = 0.033.

Correlations between the bacteriome and mycobiome were determined by drafting a matrix of Spearman correlations between the abundances of bacterial genera and fungal OTUs of the six ITS sequenced sample pairs. Abundances of the coronal and apical root segments were combined. Only bacterial genera that were present in more than 25 % of the samples and at an abundance of more than 0.05 % were included in the analysis. Only fungal OTUs that were present in more than one sample pair were included in the analysis. An FDR correction was applied to adjust the level of significance.

Statistical analyses were performed using IBM SPSS Statistics for Windows, version 21.0 (IBM Corp., Armonk, NY, USA). Diversity analyses, PCA, and PERMANOVA were performed using Paleontological Statistics (PAST) software version 3.12 [41]. Figures were drafted using GraphPad Prism 5.00 (GraphPad Software, San Diego, CA, USA). The Spearman correlation matrix was calculated in R, version 3.2.3 (A Language and Environment for Statistical Computing) using the rcorr function from the Hmisc library. Heatmaps were generated with pheatmap in R, using default settings for hierarchical clustering.

## Results

**Fig. 1:**
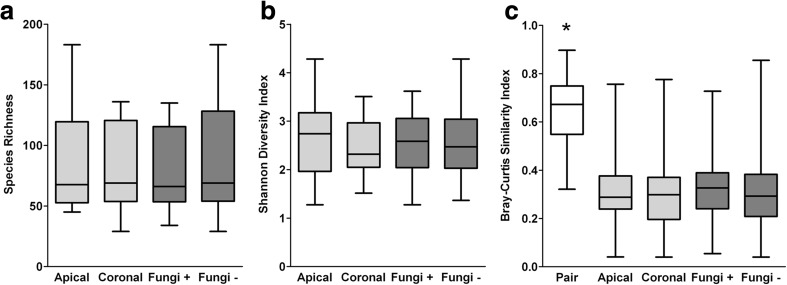
Diversity analyses of the bacteriome of root canal infections. **a** Observed species richness (number of OTUs/sample). **b** Shannon diversity index of within-sample diversity. **c** Bray-Curtis similarity index of between-sample diversity. For analysis of the species richness and Shannon diversity index, Wilcoxon signed-rank test compared apical (*N* = 22) to coronal root segments (*N* = 22; light gray) and the Mann-Whitney *U* test compared fungi positive (*N* = 21) to fungi negative root segments (*N* = 24; dark gray). For analysis of the Bray-Curtis similarity index, the Kruskal-Wallis test compared the paired root samples to the apical and coronal root segments as well as to the fungi-positive and fungi-negative root segments, with post hoc analysis using the Mann-Whitney *U* test and an FDR correction (q* = 0.033). *p < 0.001

**Fig. 2:**
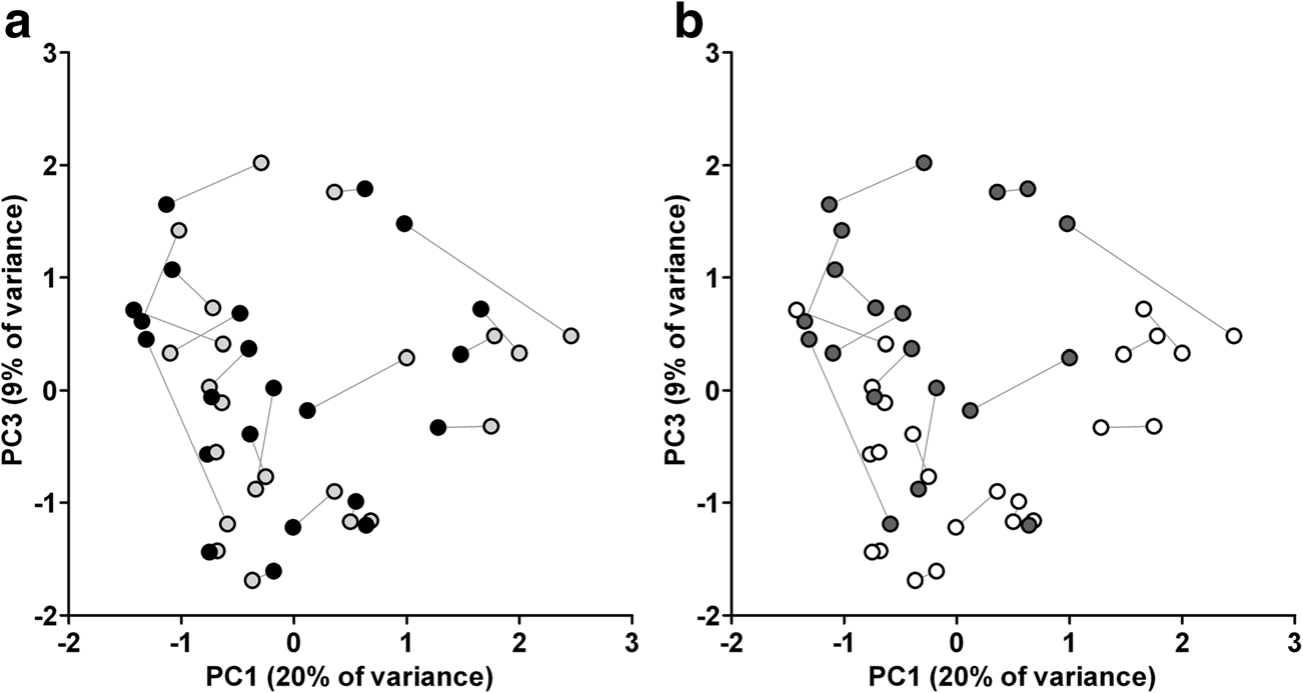
A two-dimensional ordination by principal component analysis (PCA) of the bacteriomes. **a** The bacterial composition of apical (*N* = 22; light gray) and coronal root segments (*N* = 22; black) were not statistically significantly different (*p* = 0.899, one-way permutational multivariate analysis of variance; PERMANOVA). **b** The bacterial composition of fungi-positive (*N* = 20; dark gray) and fungi-negative root segments (*N* = 24; white) was statistically significantly different (*p* = 0.008; PERMANOVA). Principal components PC1, PC2 (not shown), and PC3 explained 20, 16, and 9 % of the overall variance among samples, respectively. Corresponding apical and coronal root segments are connected with a line

A total of 26 patients contributed one tooth each. The DNA of three apical root segments could not be sequenced, because insufficient 16S rDNA could be isolated. Therefore, the corresponding three coronal root segments of the same root segment pair were not analyzed either. After processing the 16S rDNA (bacteriome) data, 622,050 reads remained in total, with 13,471 ± 4507 reads per sample. After subsampling at a depth of 6400 reads, one sample was excluded from further analyses because of too few reads. The corresponding sample of the same root segment pair was excluded from further paired analyses. A total of 338 OTUs were identified with 87 ± 41 OTUs (range 46–184) per apical root segment and 83 ± 35 OTUs (range 30–137) per coronal root segment. Apical root segments did not contain significantly more OTUs than coronal roots (*z* = −1.217, *p* = 0.224; Fig. 1a) and were similarly diverse (*z* = −1.672, *p* = 0.095; Fig. 1b). The PCA plot visualized that the bacteriome of apical and coronal root segments did not significantly differ, which was confirmed by a PERMANOVA (*F* = 0.550, *p* = 0.899; Fig. 2a). The bacteriome of the respective apical and coronal root pairs was compared to the grouped apical and coronal root segments. Root pairs were more similar than the grouped apical or coronal root segments (*H*(2) = 52.378, *p* < 0.001; Fig. 1c). The 10 most prevalent genera in all teeth were *Prevotella* (12.7 % of reads, 23/23 teeth), *Lactobacillus* (11.2 %, 21/23 teeth), *Actinomyces* (7.5 %, 23/23 teeth), *Fusobacterium* (7.2 %, 23/23 teeth), *Atopobium* (6.9 %, 21/23 teeth), *Streptococcus* (4.4 %, 23/23 teeth), *Leptotrichia* (4.3 %, 22/23 teeth), *Phocaeicola* (3.5 %, 14/23 teeth), *Pyramidobacter* (2.9 %, 6/23 teeth), and *Porphyromonas* (2.7 %, 19/23 teeth; Online Resource 1).

**Fig. 3:**
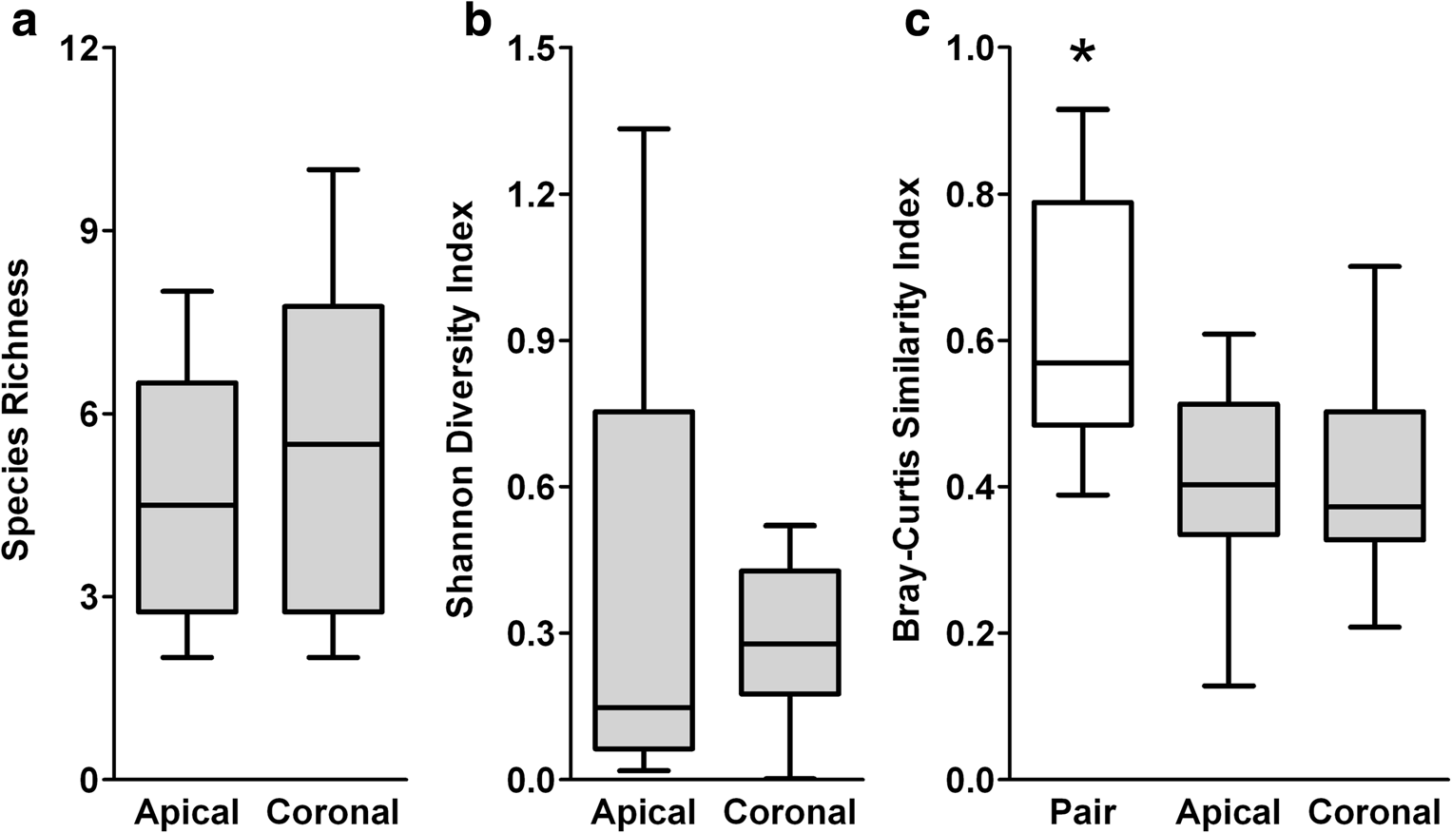
Diversity analyses of the mycobiomes of root canal infections as determined through sequencing of the ITS1 region. **a** Observed species richness (number of OTUs/sample). **b** Shannon diversity index of within-sample diversity. **c** Bray-Curtis similarity index of between-sample diversity. Wilcoxon signed-rank test was done to compare apical (*N* = 6) to coronal root segments (*N* = 6) for the species richness and Shannon diversity index. The Kruskal-Wallis test was done using the Bray-Curtis similarity indices of the paired root samples to the apical and coronal grouped root segments, with post hoc analysis using the Mann-Whitney *U* test and an FDR correction (q* = 0.033). *p < 0.001

Fungal DNA was detected in 57 % (13/23 teeth) of the teeth. The amount of fungal DNA in the samples was analyzed, using 28S rDNA qPCR, to select sample pairs for ITS sequencing. Many samples did not contain sufficient DNA for further analysis; in most cases, only the apical root segment did not contain sufficient DNA. Therefore, the ITS1 and ITS2 regions of only six root sample pairs were sequenced. Subsequent analyses describe the ITS1 results; the ITS2 results are reported in Online Resource 2. In the ITS1 analysis, 514,703 reads passed quality filtering and processing, and samples contained 39,342 ± 19,827 reads. After subsampling at a depth of 6400 reads, 28 OTUs remained. Apical root segments contained 5 ± 2 OTUs (range 2–8) and coronal root segments contained 6 ± 3 OTUs (range 2–10). Coronal root segments neither contained significantly more OTUs than apical root segments (*z* = −0.137, *p* = 0.891; Fig. 3a) nor were they more diverse (*z* = −0.105, *p* = 0.917; Fig. 3b). In the PCA, clustering of coronal and apical root segments could not be observed, which was confirmed by statistical testing (PERMANOVA, *F* = 0.327, *p* = 0.966; Online Resource 3). Similar to the bacteriome analysis, the mycobiomes of the root pairs (*N* = 6) were more similar than the grouped apical or coronal mycobiomes (*H*(2) = 7.513, *p* = 0.023; Fig. 3c). *Candida* (96.1 % of reads, 6/6 teeth) and *Malassezia* (4.1 %, 2/6 teeth) were most frequently identified (Table 1).

**Table 1:** Abundance and prevalence of fungal OTUs of root canal infections, as determined through sequencing of the ITS1 region

		All teeth (*N* = 6)		Apical samples (*N* = 6)		Coronal samples (*N* = 6)	
Phylum	Species or higher level	% Abundance	Prevalence	% Abundance	Prevalence	% Abundance	Prevalence
Ascomycota	*Ascochyta skagwayensis*	0.02	1			0.05	1
Ascomycota	Ascomycota	0.62	2	0.93	2	0.31	1
Ascomycota	*Aspergillus* (three OTUs)	0.27	4	0.26	2	0.28	2
	*- Aspergillus caesiellus*	0.17	2	0.22	1	0.11	1
	*- Aspergillus flavus*	0.08	1			0.17	1
	*- Aspergillus sydowii*	0.02	1	0.03	1		
Ascomycota	*Candida* (eight OTUs)	94.06	6	90.78	6	97.33	6
	*- Candida albicans* (two OTUs)	74.90	6	68.87	6	80.93	6
	*- Candida dubliniensis* (three OTUs)	16.72	4	18.98	4	14.45	3
	*- Candida etchellsii*	0.01	1	0.02	1		
	*- Candida glabrata*	2.14	1	2.82	1	1.47	1
Ascomycota	*Cyberlindnera jadinii*	0.01	1			0.02	1
Ascomycota	*Debaryomyces*	0.04	2	0.05	1	0.04	1
Ascomycota	Family Davidiellaceae	0.53	2	0.04	1	1.03	1
Basidiomycota	*Malassezia* (three OTUs)	4.13	2	7.87	1	0.39	2
	*- Malassezia globosa*	2.08	1	4.16	1		
	*- Malassezia restricta* (two OTUs)	2.05	2	3.70	1	0.39	2
Ascomycota	*Mycosphaerella tassiana*	0.12	2			0.25	2
Ascomycota	Order Eurotiales	0.01	1	0.01	1		
Ascomycota	*Penicillium* (two OTUs)	0.09	2	0.01	1	0.18	1
	*- Penicillium digitatum*	0.09	2	0.01	1	0.16	1
Ascomycota	*Pichia membranifaciens* (two OTUs)	0.06	1			0.11	1
Basidiomycota	*Pleurotus*	0.001	1			0.002	1
Ascomycota	*Torrubiella* sp.	0.03	2	0.05	2		
Basidiomycota	*Wallemia mellicola*	0.01	1			0.02	1

The taxon name was assigned to the most abundant sequence of the OTU, using the UNITE database. OTUs that were grouped together to the same genus are indicated by a dash

Levels of 16S rDNA did not correlate to levels of 28S rDNA (*r*_*s*_ = 0.394, *p* = 0.057), and more 16S rDNA than 28S rDNA could be retrieved from all but one sample. However, the separation between the bacteriomes of the 20 fungal positive and the 24 fungal negative root segments was statistically significant (PERMANOVA, *F* = 2.526, *p* = 0.008) and is visualized in the PCA plot (Fig. 2b). Next, the 12 ITS-sequenced segments with higher 28S rDNA loads were compared with the eight segments for which the ITS region was not sequenced since the 28S rDNA load was too low. No differences could be found between the bacteriomes of these 20 fungal positive root segments (PERMANOVA, *F* = 0.825, *p* = 0.649). Using fungal presence as class level and root segment as subclass level in LEfSe, 12 bacterial OTUs were biomarkers for the groups of the bacteriomes of root segments with and without fungi (Table 2). The four OTUs that were discriminative for bacteriomes of fungi-negative root segments contained *Fretibacterium*, *Porphyromonas*, and two *Prevotella* OTUs. The eight OTUs that were discriminative for bacteriomes of fungi-positive root segments had been assigned the following taxonomic names: *Actinomyces*, *Bifidobacterium*, four *Lactobacillus* OTUs, *Propionibacterium*, and *Streptococcus*. In post hoc analyses, all 12 OTUs were statistically significantly different between bacteriomes of root segments with and without fungi.

**Table 2:** Discriminant OTUs for groups with or without fungi as identified by linear discriminant analysis (LDA) effect size (LEfSe)

OTU nr	Taxonomy (SILVA)	Taxonomy (HOMD)	Fungal presence	Abundance (%)	Prevalence (%)	LDA effect size	*p* value (LEfSe)	*p* value (post hoc)
OTU 7	*Porphyromonas*	*Porphyromonas endodontalis*	Negative	1.4	40.0	4.1	0.015	0.015
OTU 15	*Prevotella*	*Prevotella nigrescens*	Negative	1.4	68.9	3.8	0.011	0.011
OTU 43	*Fretibacterium*	*Fretibacterium fastidiosum*	Negative	0.4	60.0	3.7	0.002	0.002
OTU 109	*Prevotella*	*Prevotella* sp. oral taxon 526	Negative	0.2	40.0	3.5	0.003	0.003
OTU 6	*Lactobacillus*	*Lactobaccilus gasseri*	Positive	3.9	46.7	4.5	0.001	0.001
OTU 9	*Lactobacillus*	*Lactobacillus paracasei*	Positive	3.0	42.2	4.3	0.005	0.005
OTU 143	*Actinomyces*	*Actinomyces* sp. oral taxon 448	Positive	0.1	46.7	4.1	0.003	0.003
OTU 33	*Lactobacillus*	*Lactobacillus vaginalis*	Positive	1.2	48.9	4.0	<0.001	<0.001
OTU 47	*Lactobacillus*	*Lactobacillus ultunensis*	Positive	0.7	48.9	4.0	0.005	0.005
OTU 253	*Streptococcus*	*Streptococcus salivarius/vestibularis*	Positive	0.1	28.9	3.8	0.001	0.001
OTU 70	*Bifidobacterium*	*Bifidobacterium dentium*	Positive	0.2	37.8	3.6	<0.001	<0.001
OTU 37	*Propionibacterium*	*Propionibacterium acidifaciens*	Positive	0.8	46.7	3.6	0.003	0.003

Post hoc analysis was performed using the Mann-Whitney *U* test, with an FDR adjusted *p* value of q* = 0.05. Taxonomy was assigned to the most abundant sequence of the OTU using the SILVA database (genus level) and the HOMD (species level)

**Fig. 4:**
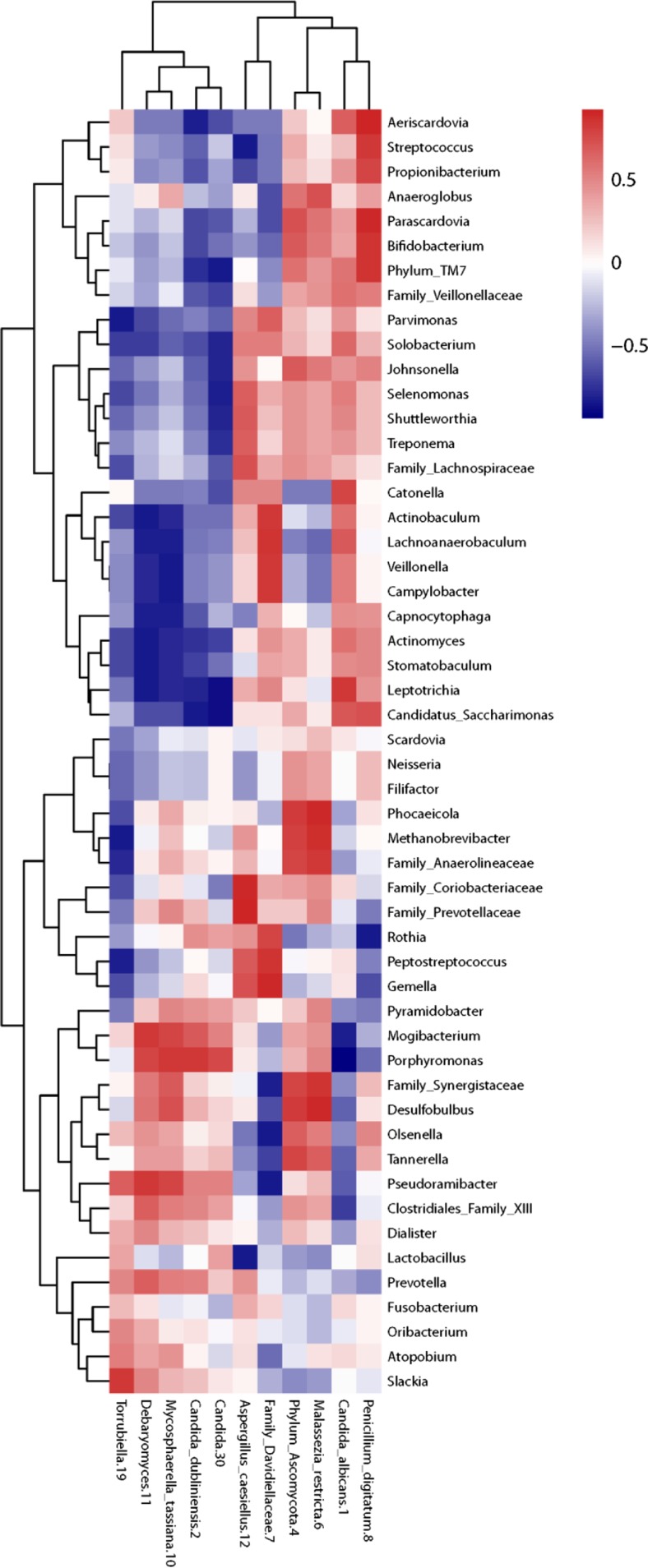
Correlations of the abundances of bacteria and fungi. A Spearman correlation matrix was drafted of bacterial genera prevalent in more than 25 % of the root pairs and at an abundance of more than 0.05 % in correlation with fungal OTUs prevalent in more than one root pair (*N* = 6). No correlations were significant after FDR correction. *Red* indicates a positive correlation; *blue* indicates a negative correlation

The Spearman correlation matrix of the bacteriome and ITS1 data revealed no significant bacteria-fungi pairs after post hoc testing. However, the fungi clustered into two groups, with mainly positive correlations for the group mostly represented by OTU 1 (*C. albicans*) and mainly negative correlations for the group mostly represented by OTU 2 (*C. dubliniensis*; Fig. 4). In contrast, the fungal cluster of OTU 2 was positively correlated to anaerobic, predominantly asaccharolytic bacteria.

## Discussion

To our knowledge, this study is the first to analyze the co-occurrence of bacteria and fungi in primary root canal infections using next-generation sequencing. Fungal presence was significantly correlated to an acidophilic bacterial composition of root canal infections.

In this study, 338 bacterial OTUs were identified, which is within the range of detected OTUs in previous studies, which identified 187 [9], 339 [14], 430 [8], 803 [12], 2168 [11], and 3253 OTUs [10]. The observed differences can be partly attributed to sampling techniques, different sequencing depths, and vast differences in processing of the sequencing reads. Among the most prevalent bacterial genera, many genera associated with endodontic disease were detected, such as *Actinomyces*, *Atopobium*, *Fusobacterium*, *Lactobacillus*, *Leptotrichia*, *Phocaeicola*, *Porphyromonas*, *Prevotella*, *Pyramidobacter*, and *Streptococcus* [7–14]. Previous research suggested a separate ecological niche for the apical and the coronal halve of the root canal [11, 42]; however, the present study was unable to confirm this. This may be caused by the limited sample size or by different ecological circumstances between the sample groups [43, 44]. The factors contributing to the ecological niche of a certain root canal system are more alike within one patient than the factors contributing to the ecological niche of a location within the root canal system, as confirmed by the analysis on the Bray-Curtis similarity indices. Driving factors can be the duration of the root canal infection [45], diet [46], intake of host-targeted drugs and antibiotics [47], and genetic variation of the host [48]. Despite the compositionally different bacteriomes of the root canal infections in this study, all bacteriomes were capable of sustaining apical periodontitis.

Although next-generation sequencing technology was applied, few fungal OTUs were identified in root canal infections, of which the main identified fungi were *Candida* and *Malassezia*. Since studies of the oral cavity only describe analysis of the ITS1 region, this article focused on the ITS1 region. Although the identified fungi in the present study are less diverse, they are in accordance with previous studies of the salivary mycobiome, where *Candida*, *Cladosporium*, *Epicoccum*, *Malassezia*, and *Saccharomyces* were most frequently detected [16, 17].

When comparing sequencing of ITS to 16S rDNA, ITS sequencing is more challenging. Firstly, fungal cells are more robust than bacterial cells, making it harder to isolate DNA. The vigorous protocol with four cycles of bead-beating with zirconia beads was proven to aid in the DNA isolation (unpublished data). In addition, DNA extraction from pulverized roots is hindered by binding of DNA to dentin, although EDTA was used to optimize the DNA isolation procedure [21]. DNA extraction is specifically complicated if DNA is present in low concentrations. In humans, fungal DNA is usually present in far lower concentrations than bacterial DNA, such as in the skin microbiome [49]. Secondly, the PCR, which is part of the sequencing procedure, has more risk of bias for ITS than for 16S rDNA. This is due to length variation in ITS regions [50] and less conserved flanking areas of the ITS regions, which complicates the development of primers that can universally amplify fungi [51]. Thirdly, classifying the fungi is complex since there is no consensus in fungal nomenclature, with different names for asexual and sexual forms of the same fungus, synonyms and misclassifications, and contamination of databases with environmental sequences [52]. For these last two reasons, it is suggested to sequence both ITS regions [51]. The present study showed a reasonable correlation between the two regions. The most prevalent species were similar (identical on genus level) in both analyses, but the low number of samples does not permit further comparison concerning the generated species richness and diversity.

This study confirms the co-occurrence of acidogenic bacteria and fungi, which is in accordance with studies on saliva in which an increase in oral *Candida* load is linked to an acidogenic and less diverse bacteriome [53, 54]. The cross-sectional nature of the present study does not allow conclusions to be drawn on whether acidogenic bacteria and fungi reside in root canal infections because the ecological niches simply overlap or if they have an ecological relationship. Possibly, as the bacterial load increases, bacteria could create a more suitable environment for fungi. Bacteria can more easily invade dentin and degrade it [55, 56]. Thereby, they may facilitate fungal colonization. Although the correlation between loads could not be confirmed within this study, such a correlation could be confirmed in studies that use more samples and collect data on the duration of the infection. In addition, a comparison of the microbial profile of endodontic infections to deep dentin caries can give suggestions about the relation between bacteria and fungi. Deep dentin caries mainly contains lactobacilli, streptococci, *Bifidobacterium*, *Prevotella*, and *Candida*, which all participate in dentin degradation [56]. This profile is very similar to the main microorganisms identified within the root canal infections of this study, and especially to the bacteriome of fungal-positive root segments. Fungi were most prevalent in the coronal halve of the root canal, close to the saccharolytic and acidogenic bacteria of a possible caries cavity and the influx of carbohydrates from the oral cavity. Despite this hypothetical relation with fungal presence, this study was unable to confirm the presence of more acidogenic bacteria in the coronal root segments. Cariogenic degradation of the root canal is also rarely observed. The absence of a true cariogenic niche is supported by the lack of an ecological shift toward less diversity, as observed when the pH drops significantly [57]. However, this might be caused by statistical limitations of the relatively small sample size [44], the limited availability of carbohydrate nutrients within the root canal system, the buffering by dentin [58], or lactate consumption by *Candida* [59]. Contrary to a possible synergism between acidogenic bacteria and *Candida*, some lactobacilli have antifungal properties and are often used as probiotics against *C. albicans* [60]. For instance, *L. paracasei* was found to inhibit *C. albicans*. It was not determined what caused this inhibition. However, a low pH, bacteriocin, and H_2_O_2_ production are suggested mechanisms [60]. Further research should explain the co-occurrence of fungi and acidogenic bacteria in root canal infections.

The possible effect of bacterial-fungal co-occurrence on the disease process and treatment success is still unclear. In analogous infections, the presence of fungi in addition to bacteria is known to complicate the infection and reduce treatment success. In a mouse model, coinfection of *C. albicans* and *Streptococcus oralis* or *Staphylococcus aureus* can enhance their virulence leading to increased oral thrush and deep organ infection [18, 19]. Interaction between *C. albicans* and *S. aureus* or *Staphylococcus epidermidis* inhibits the effect of antibiotics and antifungals and results in less effective treatment in vitro [61, 62]. In mice, *C. albicans* can only colonize the gut after the microbiome is perturbed by antibiotics administration. After recovery, diversity was not affected, but different bacteria were present [63]. The effect on health of this altered microbiome is still unclear. As fungi are morphologically and physiologically very different from bacteria, they may also require different antimicrobial treatment when detected in root canal infections.

In conclusion, the present study clearly indicates a correlation of the root canal infection mycobiome with the bacteriome. However, it is still uncertain whether the co-occurrence of bacteria and fungi affects root canal infections and treatment success. Therefore, further research should study the interaction between bacteria and fungi in root canal infections and whether fungi influence treatment success. If so, alternative treatment strategies should be explored.

## Electronic supplementary material

ESM 1 (PDF 128 kb)

ESM 2 (PDF 82 kb)

ESM 3 (PDF 197 kb)

## References

[1] Kakehashi S, Stanley HR, Fitzgerald RJ (1965). The effects of surgical exposures of dental pulps in germ-free and conventional laboratory rats. Oral Surg Oral Med Oral Pathol.

[2] Matsumiya S, Kitamura M (1960). Histopathological and histobacteriological studies of the relation between the condition of sterilization of the interior of the root canal and the healing process of periapical tissues in experimentally infected root canal treatment. Bull Tokyo Dent Coll.

[3] Wolf TG, Paqué F, Zeller M (2016). Root canal morphology and configuration of 118 mandibular first molars by means of micro–computed tomography: an ex vivo study. J Endod.

[4] Ricucci D, Siqueira JF (2010). Biofilms and apical periodontitis: study of prevalence and association with clinical and histopathologic findings. J Endod.

[5] Sundqvist G, Figdor D, Persson S (1998). Microbiologic analysis of teeth with failed endodontic treatment and the outcome of conservative re-treatment. Oral Surg Oral Med Oral Pathol Oral Radiol Endod.

[6] Munson MA, Pitt-Ford T, Chong B (2002). Molecular and cultural analysis of the microflora associated with endodontic infections. J Dent Res.

[7] Li L, Hsiao WWL, Nandakumar R (2010). Analyzing endodontic infections by deep coverage pyrosequencing. J Dent Res.

[8] Santos AL, Siqueira JF, Rôças IN (2011). Comparing the bacterial diversity of acute and chronic dental root canal infections. PLoS One.

[9] Siqueira JF, Alves FR, Rôças IN (2011). Pyrosequencing analysis of the apical root canal microbiota. J Endod.

[10] Hsiao W, Li K, Liu Z (2012). Microbial transformation from normal oral microbiota to acute endodontic infections. BMC Genomics.

[11] Özok AR, Persoon IF, Huse SM (2012). Ecology of the microbiome of the infected root canal system: a comparison between apical and coronal root segments. Int Endod J.

[12] Hong B-Y, Lee T-K, Lim S-M (2013). Microbial analysis in primary and persistent endodontic infections by using pyrosequencing. J Endod.

[13] Vengerfeldt V, Spilka K, Saag M (2014). Highly diverse microbiota in dental root canals in cases of apical periodontitis (data of Illumina sequencing). J Endod.

[14] Tzanetakis GN, Azcarate-Peril MA, Zachaki S (2015). Comparison of bacterial community composition of primary and persistent endodontic infections using pyrosequencing. J Endod.

[15] Egan MW, Spratt DA, Ng YL (2002). Prevalence of yeasts in saliva and root canals of teeth associated with apical periodontitis. Int Endod J.

[16] Ghannoum MA, Jurevic RJ, Mukherjee PK (2010). Characterization of the oral fungal microbiome (mycobiome) in healthy individuals. PLoS Pathog.

[17] Dupuy AK, David MS, Li L (2014). Redefining the human oral mycobiome with improved practices in amplicon-based taxonomy: discovery of *Malassezia* as a prominent commensal. PLoS One.

[18] Xu H, Sobue T, Thompson A (2014). Streptococcal co-infection augments *Candida* pathogenicity by amplifying the mucosal inflammatory response. Cell Microbiol.

[19] Schlecht LM, Peters BM, Krom BP (2015). Systemic *Staphylococcus aureus* infection mediated by *Candida albicans* hyphal invasion of mucosal tissue. Microbiology.

[20] Akpata ES (1976). Effect of endodontic procedures on the population of viable microorganisms in the infected root canal. J Endod.

[21] Brundin M, Figdor D, Johansson A (2014). Preservation of bacterial DNA by human dentin. J Endod.

[22] Ciric L, Pratten J, Wilson M (2010). Development of a novel multi-triplex qPCR method for the assessment of bacterial community structure in oral populations. Environ Microbiol Rep.

[23] Vollmer T, Störmer M, Kleesiek K (2008). Evaluation of novel broad-range real-time PCR assay for rapid detection of human pathogenic fungi in various clinical specimens. J Clin Microbiol.

[24] Herlemann DPR, Labrenz M, Jurgens K (2011). Transitions in bacterial communities along the 2000 km salinity gradient of the Baltic Sea. ISME J.

[25] Caporaso JG, Lauber CL, Walters WA (2011). Global patterns of 16S rRNA diversity at a depth of millions of sequences per sample. Proc Natl Acad Sci U S A.

[26] Kozich JJ, Westcott SL, Baxter NT (2013). Development of a dual-index sequencing strategy and curation pipeline for analyzing amplicon sequence data on the MiSeq Illumina sequencing platform. Appl Environ Microbiol.

[27] White TJ, Bruns T, Lee SJWT, Innis MA, Gelfand DH, Sninsky JJ, White TJ (1990). Amplification and direct sequencing of fungal ribosomal RNA genes for phylogenetics. PCR protocols: a guide to methods and applications, vol 18.

[28] Gardes M, Bruns TD (1993). ITS primers with enhanced specificity for basidiomycetes--application to the identification of mycorrhizae and rusts. Mol Ecol.

[29] Turenne CY, Sanche SE, Hoban DJ (1999). Rapid identification of fungi by using the ITS2 genetic region and an automated fluorescent capillary electrophoresis system. Clin Microbiol.

[30] DeAngelis MM, Wang DG, Hawkins TL (1995). Solid-phase reversible immobilization for the isolation of PCR products. Nucleic Acids Res.

[31] Edgar RC, Flyvbjerg H (2015). Error filtering, pair assembly and error correction for next-generation sequencing reads. Bioinformatics.

[32] Edgar RC (2013). UPARSE: highly accurate OTU sequences from microbial amplicon reads. Nat Methods.

[33] Caporaso JG, Kuczynski J, Stombaugh J (2010). QIIME allows analysis of high-throughput community sequencing data. Nat Methods.

[34] Cole JR, Wang Q, Cardenas E (2009). The ribosomal database project: improved alignments and new tools for rRNA analysis. Nucleic Acids Res.

[35] Quast C, Pruesse E, Yilmaz P (2013). The SILVA ribosomal RNA gene database project: improved data processing and web-based tools. Nucleic Acids Res.

[36] Chen T, Yu W-H, Izard J et al (2010) The human oral microbiome database: a web accessible resource for investigating oral microbe taxonomic and genomic information. Database 2010. doi:10.1093/database/baq013 10.1093/database/baq013 PMC2911848 20624719

[37] Bengtsson-Palme J, Ryberg M, Hartmann M (2013). Improved software detection and extraction of ITS1 and ITS2 from ribosomal ITS sequences of fungi and other eukaryotes for analysis of environmental sequencing data. Methods Ecol Evol.

[38] Kõljalg U, Nilsson RH, Abarenkov K (2013). Towards a unified paradigm for sequence-based identification of fungi. Mol Ecol.

[39] Segata N, Izard J, Waldron L (2011). Metagenomic biomarker discovery and explanation. Genome Biol.

[40] Benjamini Y, Hochberg Y (1995). Controlling the false discovery rate: a practical and powerful approach to multiple testing. J Roy Stat Soc B Met.

[41] Hammer Ø, Harper D, Ryan P (2001). PAST: paleontological statistics software package for education and data analysis. Palaeontol Electron.

[42] Alves FR, Siqueira JF, Carmo FL (2009). Bacterial community profiling of cryogenically ground samples from the apical and coronal root segments of teeth with apical periodontitis. J Endod.

[43] Sundqvist G (1976). Bacteriological studies of necrotic dental pulps (PhD dissertation).

[44] Kelly BJ, Gross R, Bittinger K (2015). Power and sample-size estimation for microbiome studies using pairwise distances and PERMANOVA. Bioinformatics.

[45] Fabricius L, Dahlén G, Öhman AE (1982). Predominant indigenous oral bacteria isolated from infected root canals after varied times of closure. Scand J Dent Res.

[46] David LA, Maurice CF, Carmody RN (2014). Diet rapidly and reproducibly alters the human gut microbiome. Nature.

[47] Maurice CF, Haiser Henry J, Turnbaugh Peter J (2013). Xenobiotics shape the physiology and gene expression of the active human gut microbiome. Cell.

[48] Blekhman R, Goodrich JK, Huang K (2015). Host genetic variation impacts microbiome composition across human body sites. Genome Biol.

[49] Gao Z, Perez-Perez GI, Chen Y (2010). Quantitation of major human cutaneous bacterial and fungal populations. J Clin Microbiol.

[50] Manter DK, Vivanco JM (2007). Use of the ITS primers, ITS1F and ITS4, to characterize fungal abundance and diversity in mixed-template samples by qPCR and length heterogeneity analysis. J Microbiol Methods.

[51] Op De Beeck M, Lievens B, Busschaert P (2014). Comparison and validation of some ITS primer pairs useful for fungal metabarcoding studies. PLoS One.

[52] Dollive S, Peterfreund GL, Sherrill-Mix S (2012). A tool kit for quantifying eukaryotic rRNA gene sequences from human microbiome samples. Genome Biol.

[53] Kraneveld EA, Buijs MJ, Bonder MJ (2012). The relation between oral *Candida* load and bacterial microbiome profiles in Dutch older adults. PLoS One.

[54] O'Donnell LE, Robertson D, Nile CJ (2015). The oral microbiome of denture wearers is influenced by levels of natural dentition. PLoS One.

[55] Waltimo TMT, Ørstavik D, Sirén EK (2000). In vitro yeast infection of human dentin. J Endod.

[56] Simón-Soro Á, Belda-Ferre P, Cabrera-Rubio R (2013). A tissue-dependent hypothesis of dental caries. Caries Res.

[57] Pham LC, van Spanning RJ, Röling WF (2009). Effects of probiotic *Lactobacillus salivarius* W24 on the compositional stability of oral microbial communities. Arch Oral Biol.

[58] Camps J, Pashley DH (2000). Buffering action of human dentin in vitro. J Adhes Dent.

[59] Willems HM, Kos K, Jabra-Rizk MA et al (2016) *Candida albicans* In oral biofilms could prevent caries. Pathog Dis 74. doi:10.1093/femspd/ftw039 10.1093/femspd/ftw039 27129365

[60] Hasslöf P, Hedberg M, Twetman S (2010). Growth inhibition of oral mutans streptococci and *Candida* by commercial probiotic lactobacilli - an in vitro study. BioMed Central.

[61] Harriott MM, Noverr MC (2010). Ability of *Candida albicans* mutants to induce *Staphylococcus aureus* vancomycin resistance during polymicrobial biofilm formation. Antimicrob Agents Chemother.

[62] Adam B, Baillie GS, Douglas LJ (2002). Mixed species biofilms of *Candida albicans* and *Staphylococcus epidermidis*. J Med Microbiol.

[63] Erb Downward JR, Falkowski NR, Mason KL (2013). Modulation of post-antibiotic bacterial community reassembly and host response by *Candida albicans*. Sci Rep.

