# Supplementary material for: The mycobiome of root canal infections is correlated to the bacteriome
Source: Clin Oral Investig. 2016 Oct 22;21(5):1871–81. doi: 10.1007/s00784-016-1980-3 (PMC5442261; doi:10.1007/s00784-016-1980-3)
Supplement: Supplementary file 1 — (PDF 128 kb) [file 784_2016_1980_MOESM1_ESM.pdf]

The mycobiome of root canal infections is correlated to the bacteriome. *Clinical Oral Investigations*

Ilona F. Persoon[1], Mark J. Buijs[1], Ahmet R. Özok[2], Wim Crielaard[1], Bastiaan P. Krom[1], Egija Zaura[1], Bernd W. Brandt[1]
Affiliations:[1]Department of Preventive Dentistry; [2]Department of Endodontology, Academic Centre for Dentistry Amsterdam (ACTA), University of Amsterdam and Vrije Universiteit Amsterdam, The Netherlands
Correspondence: Ilona F. Persoon, i.persoon@acta.nl

Table Abundance and prevalence of bacterial genera of root canal infections

		All teeth (N = 23)		Apical samples (N = 22)		Coronal samples (N = 23)	
Phylum	Genus or higher level	% Abundance	Prevalence	% Abundance	Prevalence	% Abundance	Prevalence
Bacteroidetes	*Prevotella*	12.74	23	13.08	22	12.42	22
Firmicutes	*Lactobacillus*	11.18	21	7.41	16	14.79	19
Actinobacteria	*Actinomyces*	7.47	23	4.39	21	10.42	23
Fusobacteria	*Fusobacterium*	7.20	23	9.33	21	5.16	22
Actinobacteria	*Atopobium*	6.94	21	4.14	19	9.61	19
Firmicutes	*Streptococcus*	4.39	23	4.62	19	4.17	23
Fusobacteria	*Leptotrichia*	4.32	22	6.56	18	2.17	20
Bacteroidetes	*Phocaeicola*	3.45	14	3.65	12	3.26	14
Synergistetes	*Pyramidobacter*	2.85	6	3.66	4	2.07	4
Bacteroidetes	*Porphyromonas*	2.68	19	3.21	15	2.18	16
Firmicutes	Clostridiales Family XIII	2.50	20	2.97	18	2.05	17
Bacteroidetes	*Alloprevotella*	2.37	17	2.21	12	2.52	12
TM7	Phylum TM7	1.92	18	1.62	13	2.21	18
Actinobacteria	*Actinobaculum*	1.72	16	1.30	13	2.12	15
Firmicutes	*Parvimonas*	1.59	22	2.24	20	0.97	19
Firmicutes	*Dialister*	1.58	21	1.88	18	1.30	21
Firmicutes	*Pseudoramibacter*	1.57	19	1.28	15	1.86	17
Actinobacteria	*Corynebacterium*	1.53	17	1.20	12	1.83	12
Firmicutes	*Veillonella*	1.42	21	1.64	18	1.21	21
Firmicutes	*Peptostreptococcus*	1.41	12	1.98	11	0.87	12
Actinobacteria	*Olsenella*	1.38	21	1.39	18	1.36	20
Firmicutes	*Filifactor*	1.38	16	1.45	14	1.31	14
Firmicutes	*Anaeroglobus*	1.29	16	1.81	13	0.79	12
Firmicutes	*Selenomonas*	1.24	23	1.42	21	1.07	20
Actinobacteria	*Propionibacterium*	1.16	16	1.33	13	1.00	16
Spirochaetae	*Treponema*	1.04	19	1.41	17	0.70	15
Euryarchaeota	*Methanobrevibacter*	1.02	11	0.79	8	1.23	9
Proteobacteria	*Campylobacter*	0.89	20	1.35	18	0.44	18
Proteobacteria	*Lautropia*	0.83	9	0.29	7	1.35	9
Firmicutes	*Oribacterium*	0.83	20	1.24	19	0.43	18
Firmicutes	*Mogibacterium*	0.81	20	1.23	18	0.42	18
Bacteroidetes	*Tannerella*	0.71	19	0.96	16	0.47	15
Actinobacteria	*Rothia*	0.61	20	0.21	15	1.00	17
Synergistetes	Family Synergistaceae	0.41	18	0.48	16	0.35	14
Synergistetes	*Fretibacterium*	0.38	16	0.40	14	0.36	13
Actinobacteria	*Scardovia*	0.38	18	0.21	12	0.54	15
Actinobacteria	*Parascardovia*	0.34	14	0.43	9	0.26	11
Chloroflexi	Family Anaerolineaceae	0.32	12	0.33	10	0.30	10
Bacteroidetes	*Capnocytophaga*	0.29	12	0.32	10	0.27	9
Firmicutes	Family Veillonellaceae	0.24	18	0.27	14	0.21	15
Bacteroidetes	RC9 gut group	0.22	12	0.28	10	0.17	12
Actinobacteria	*Bifidobacterium*	0.21	12	0.09	7	0.32	10
Firmicutes	*Shuttleworthia*	0.20	16	0.25	11	0.15	13
Firmicutes	*Lachnoanaerobaculum*	0.19	13	0.26	12	0.13	12

Actinobacteria	Family Coriobacteriaceae	0.18	9	0.07	6	0.29	7
Firmicutes	Family Lachnospiraceae	0.14	11	0.24	10	0.04	10
Proteobacteria	*Neisseria*	0.14	13	0.07	10	0.21	12
Actinobacteria	*Slackia*	0.14	19	0.14	15	0.14	16
Firmicutes	*Howardella*	0.13	10	0.23	6	0.04	6
Proteobacteria	*Haemophilus*	0.12	12	0.12	7	0.11	9
Actinobacteria	Family Corynebacteriaceae	0.11	2	0.21	1	0.01	2
Firmicutes	*Johnsonella*	0.11	15	0.13	11	0.08	13
Firmicutes	*Solobacterium*	0.10	18	0.12	15	0.08	8
Firmicutes	Lactobacillales familiy P5D1-392	0.10	15	0.09	8	0.11	11
Proteobacteria	*Aggregatibacter*	0.09	9	0.13	6	0.06	6
Proteobacteria	*Desulfobulbus*	0.09	13	0.08	11	0.09	11
Firmicutes	*Catonella*	0.07	10	0.06	8	0.07	7
Proteobacteria	*Cardiobacterium*	0.06	6	0.07	5	0.04	6
Bacteroidetes	*Proteiniphilum*	0.06	2	0.11	2	0.01	2
Firmicutes	*Abiotrophia*	0.05	7	0.03	6	0.07	5
Bacteroidetes	Family Prevotellaceae	0.05	9	0.02	6	0.09	7
Firmicutes	*Gemella*	0.05	11	0.07	10	0.03	9
Firmicutes	*Peptococcus*	0.05	10	0.07	7	0.03	9
Actinobacteria	*Cryptobacterium*	0.05	11	0.06	9	0.03	8
Firmicutes	*Fastidiosipila*	0.04	2	0.08	2	0.01	2
Bacteroidetes	Family WCHB1-69	0.04	8	0.06	6	0.02	7
Firmicutes	*Stomatobaculum*	0.04	12	0.04	9	0.04	5
Firmicutes	Family Erysipelotrichaceae	0.04	8	0.04	5	0.03	7
TM7	Candidatus Saccharimonas	0.04	9	0.06	8	0.01	5
Bacteroidetes	*Paludibacter*	0.03	10	0.04	6	0.03	8
Bacteroidetes	*Bacteroides*	0.03	5	0.06	3	0.01	4
Tenericutes	*Mycoplasma*	0.03	5	0.04	5	0.03	1
Firmicutes	*Bulleidia*	0.03	5	0.05	4	0.02	3
Firmicutes	Family Defluviitaleaceae	0.03	6	0.06	6	0.01	4
Proteobacteria	*Pelomonas*	0.03	9	0.05	8	0.01	3
Bacteroidetes	Bacteroidales family S24-7	0.03	1	0.05	1	0.01	1
Bacteroidetes	*Odoribacter*	0.03	2	0.06	2	<0.01	2
Actinobacteria	*Aeriscardovia*	0.03	4	0.01	2	0.04	4
Actinobacteria	*Enterorhabdus*	0.03	9	0.02	6	0.03	8
Actinobacteria	Phylum Actinobacteria	0.03	4	0.02	2	0.03	4
Firmicutes	Clostridiales Family XI	0.02	4	<0.01	1	0.05	3
Fusobacteria	Family Leptotrichiaceae	0.02	5	0.03	3	0.02	4
Firmicutes	*Eggerthia*	0.02	5	0.03	4	0.01	4
Firmicutes	Family Peptostreptococcaceae	0.02	7	0.03	7	<0.01	2
Proteobacteria	*Pseudomonas*	0.02	6	0.02	3	0.01	5
Proteobacteria	*Bradyrhizobium*	0.02	8	0.02	6	0.01	5
Firmicutes	*Megasphaera*	0.02	9	0.02	6	0.01	6
Firmicutes	Family Ruminococcaceae	0.02	7	0.03	6	<0.01	2
Bacteroidetes	*Flavobacterium*	0.02	1	0.03	1		
Proteobacteria	*Kingella*	0.02	8	0.02	5	0.01	5
Firmicutes	*Pediococcus*	0.01	3	0.01	1	0.02	3
Bacteroidetes	*Bergeyella*	0.01	5	0.02	5	0.01	2
Proteobacteria	*Eikenella*	0.01	8	0.02	6	<0.01	3
Proteobacteria	*Methylobacterium*	0.01	6	0.02	5	<0.01	1
Proteobacteria	*Sphingomonas*	0.01	7	0.01	5	0.01	3
Proteobacteria	*Comamonas*	0.01	3	0.02	3	<0.01	1
Firmicutes	Family Bacillaceae	0.01	6	0.01	5	<0.01	3
Proteobacteria	*Tepidimonas*	0.01	6	<0.01	3	0.01	3
Proteobacteria	*Paracoccus*	0.01	2	0.01	1	<0.01	1
Firmicutes	*Coprococcus*	0.01	3	0.01	3	<0.01	2
Tenericutes	*Acholeplasma*	0.01	3	0.01	3	<0.01	1

Firmicutes	Clostridiales vadinBB60 group	0.01	4	0.01	3	0.01	4
Bacteroidetes	*Sediminibacterium*	0.01	3	0.01	3		
Firmicutes	*Butyrivibrio*	0.01	2	0.01	2	<0.01	2
Spirochaetae	*Spirochaeta*	0.01	3	0.01	3	<0.01	2
Proteobacteria	*Burkholderia*	<0.01	3	<0.01	1	0.01	3
Proteobacteria	*Caulobacter*	<0.01	4	<0.01	2	0.01	2
SR1	Phylum SR1	<0.01	5	0.01	4	<0.01	2
Proteobacteria	*Acinetobacter*	<0.01	2	0.01	1	<0.01	1
Proteobacteria	Family Pasteurellaceae	<0.01	5	<0.01	4	<0.01	2
Tenericutes	Order NB1-n	<0.01	3	0.01	3	<0.01	1
Firmicutes	*Christensenella*	<0.01	1	0.01	1		
Proteobacteria	*Sphingobium*	<0.01	2	<0.01	2		
Proteobacteria	*Massilia*	<0.01	1	<0.01	1		
Bacteroidetes	*Pedobacter*	<0.01	1	<0.01	1		
Actinobacteria	*Nitriliruptor*	<0.01	4	<0.01	3	<0.01	2
Proteobacteria	*Novosphingobium*	<0.01	2	<0.01	2	<0.01	1
Actinobacteria	*Jatrophihabitans*	<0.01	2	<0.01	1	<0.01	1
Proteobacteria	*Klebsiella*	<0.01	2	<0.01	2		
Proteobacteria	*Propionivibrio*	<0.01	2			<0.01	2
Proteobacteria	Family Phyllobacteriaceae	<0.01	2	<0.01	2		
Bacteroidetes	*Cytophaga*	<0.01	1	<0.01	1		
Proteobacteria	Family Solimonadaceae	<0.01	1	<0.01	1		
Bacteroidetes	Phylum Bacteroidetes	<0.01	1			<0.01	1
Proteobacteria	*Sorangium*	<0.01	1	<0.01	1		
Proteobacteria	*Curvibacter*	<0.01	3	<0.01	2	<0.01	1
Proteobacteria	*Aquabacterium*	<0.01	2	<0.01	2	<0.01	1
Proteobacteria	*Serratia*	<0.01	2	<0.01	2		
Proteobacteria	*Camelimonas*	<0.01	1	<0.01	1		
Cyanobacteria	*Chroococcidiopsis*	<0.01	1	<0.01	1		
Firmicutes	*Dolosigranulum*	<0.01	1	<0.01	1		
Bacteroidetes	Family p-2534-18B5 gut group	<0.01	1	<0.01	1		
Firmicutes	*Pelospora*	<0.01	1	<0.01	1		
Proteobacteria	Family Caulobacteraceae	<0.01	2	<0.01	2		
Bacteroidetes	*Chryseobacterium*	<0.01	1	<0.01	1		
Proteobacteria	*Desulfomicrobium*	<0.01	1			<0.01	1
Firmicutes	*Lactococcus*	<0.01	1	<0.01	1		
Actinobacteria	Family Propionibacteriaceae	<0.01	2			<0.01	2
Proteobacteria	*Alkanibacter*	<0.01	1	<0.01	1		
Proteobacteria	Family GR-WP33-58	<0.01	1	<0.01	1		
Proteobacteria	*Xylella*	<0.01	1	<0.01	1		
Proteobacteria	*Brevundimonas*	<0.01	1	<0.01	1		
Proteobacteria	Family Enterobacteriaceae	<0.01	1			<0.01	1
Proteobacteria	*Halomonas*	<0.01	1	<0.01	1		
Actinobacteria	*Micrococcus*	<0.01	1			<0.01	1
Firmicutes	*Staphylococcus*	<0.01	1			<0.01	1
Proteobacteria	*Stenotrophomonas*	<0.01	1	<0.01	1		
