# Supplementary material for: The mycobiome of root canal infections is correlated to the bacteriome
Source: Clin Oral Investig. 2016 Oct 22;21(5):1871–81. doi: 10.1007/s00784-016-1980-3 (PMC5442261; doi:10.1007/s00784-016-1980-3)
Supplement: Supplementary file 2 — (PDF 82 kb) [file 784_2016_1980_MOESM2_ESM.pdf]

The mycobiome of root canal infections is correlated to the bacteriome. *Clinical Oral Investigations*
Ilona F. Persoon[1], Mark J. Buijs[1], Ahmet R. Özok[2], Wim Crielaard[1], Bastiaan P. Krom[1], Egija Zaura[1], Bernd W. Brandt[1]
Affiliations:[1]Department of Preventive Dentistry; [2]Department of Endodontology, Academic Centre for Dentistry Amsterdam (ACTA), University of Amsterdam and Vrije Universiteit Amsterdam, The Netherlands
Correspondence: Ilona F. Persoon, i.persoon@acta.nl

Results of ITS2 sequencing

In the ITS2 analysis, 482 693 reads passed quality filtering and processing, with 40 173 ± 12 335 reads per sample (range 4831 – 56 874). Samples were normalized by subsampling at a depth of 4800 reads, after which 23 OTUs remained (Table ESM 2). Apical root segments contained 3 ± 2 OTUs (range 1 – 5), while coronal root segments contained 5 ± 4 OTUs (range 1 - 11). Coronal root segments did not contain more OTUs (z = -0.213, *p* = 0.832; Fig. ESM 2.1a) and were not more diverse (z = -0.314, *p* = 0.753; Fig. ESM 2.1b) than apical segments. Analysis of Bray-Curtis similarity indices revealed no distinct grouping (H(2) = 4.054, *p* = 0.132; Fig. ESM 2.1c). In the PCA, coronal and apical root segments were not significantly different either (PERMANOVA, F = 0.222, *p* = 0.987; Fig. ESM 2.2).

Table ESM 2 Abundance and prevalence of fungal OTUs of root canal infections, as determined through sequencing of the ITS2 region

		All teeth (N = 6)		Apical samples (N = 6)		Coronal samples (N = 6)	
**Phylum**	**Species or higher level**	**% Abundance**	**Prevalence**	**% Abundance**	**Prevalence**	**% Abundance**	**Prevalence**
Ascomycota	*Aspergillus*	0.22	1			0.51	1
Ascomycota	*Candida* (7 OTUs)	80.13	6	93.69	6	96.73	6
	Candida albicans (3 OTUs)	60.95	6	68.91	6	75.91	6
	Candida dubliniensis (2 OTUs)	12.75	2	16.45	2	13.85	2
	Candida glabrata	6.43	1	8.33	1	6.96	1
	Candida zeylanoides	0.001	1			0.003	1
Ascomycota	*Citeromyces matritensis*	0.10	1	0.23	1		
Basidiomycota	*Cryptococcus neoformans*	0.11	2	0.18	1	0.08	1
Ascomycota	*Debaryomyces*	0.74	2	0.08	1	1.68	2
Ascomycota	*Exophiala oligosperma*	0.01	1			0.03	1
Ascomycota	*Lecanicillium*	0.29	1	0.68	1		
Basidiomycota	*Malassezia* (4 OTUs)	2.16	3	4.83	2	0.29	1
	Malassezia globosa (2 OTUs)	1.03	3	2.44	2	0.02	1
	Malassezia restricta	1.05	2	2.39	1	0.11	1
	Malassezia sp	0.07	1			0.16	1
Ascomycota	Order Capnodiales	0.001	1			0.003	1
Ascomycota	*Penicillium digitatum*	0.14	2	0.12	1	0.21	2
Ascomycota	*Phoma saxea*	0.09	1			0.21	1
Ascomycota	Phylum Ascomycota (2 OTUs)	0.11	1			0.25	1
Ascomycota	*Pichia fermentans*	0.01	1	0.01	1		
Ascomycota	*Saccharomyces*	0.07	1	0.16	1		

Taxonomy was assigned to the most abundant sequence of the clustered sequences of the OTU, clustered at 97% similarity, using the UNITE database. OTUs grouped together belonging to one genus are depicted in grey

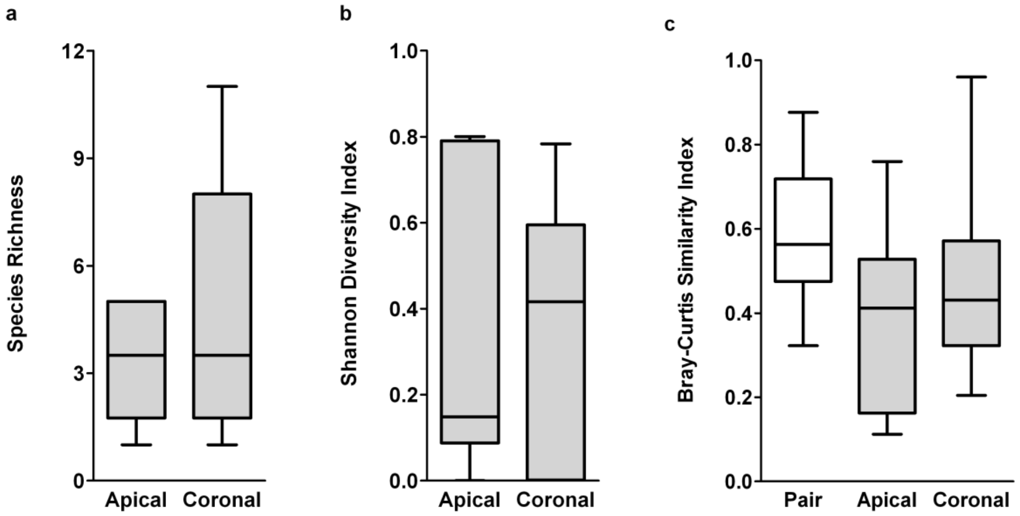

Fig. ESM 2.1 Diversity analyses of the mycobiomes of root canal infections as determined through sequencing of the ITS2 region. **a** observed species richness (number of OTUs / sample). **b** Shannon diversity index of within-sample diversity. **c** Bray-Curtis similarity index of between-sample diversity. For analysis of the species richness and Shannon diversity index, Wilcoxon signed-rank test compared apical (N = 6) to coronal root segments (N = 6). For analysis of the Bray-Curtis similarity index, the Kruskal-Wallis test compared the paired root samples to the apical and coronal root segments. No significant relations were found

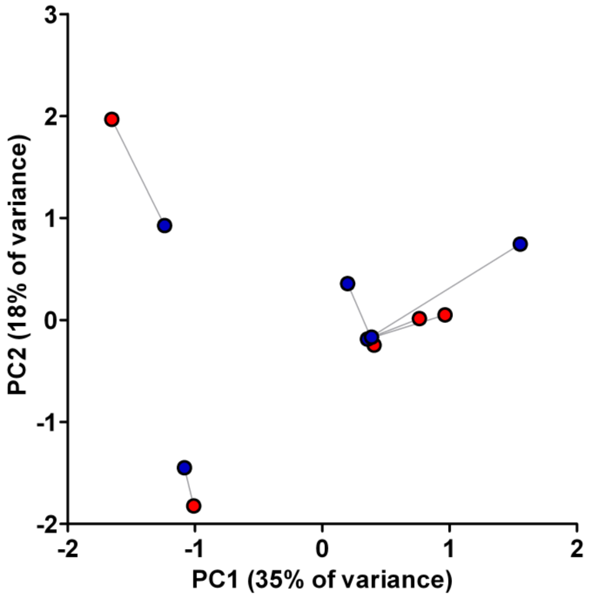

Fig. ESM 2.2 A two-dimensional ordination by principal component analysis (PCA) of the mycobiome as determined through ITS2 sequencing. The apical (N = 6; red) and coronal root segments (N = 6; blue) were not statistically significantly different (F = 0.222, p = 0.987, PERMANOVA). Principal components PC1, PC2 and PC3 (not shown) explained 35%, 18% and 17% of the overall variance among samples, respectively. Corresponding apical and coronal root segments are connected with a line
