# Supplementary material for: The mycobiome of root canal infections is correlated to the bacteriome
Source: Clin Oral Investig. 2016 Oct 22;21(5):1871–81. doi: 10.1007/s00784-016-1980-3 (PMC5442261; doi:10.1007/s00784-016-1980-3)
Supplement: Supplementary file 3 — (PDF 197 kb) [file 784_2016_1980_MOESM3_ESM.pdf]

The mycobiome of root canal infections is correlated to the bacteriome. *Clinical Oral Investigations*

Ilona F. Persoon[1], Mark J. Buijs[1], Ahmet R. Özok[2], Wim Crielaard[1], Bastiaan P. Krom[1], Egija Zaura[1], Bernd W. Brandt[1]
Affiliations:[1]Department of Preventive Dentistry; [2]Department of Endodontology, Academic Centre for Dentistry Amsterdam (ACTA), University of Amsterdam and Vrije Universiteit Amsterdam, The Netherlands
Correspondence: Ilona F. Persoon, i.persoon@acta.nl

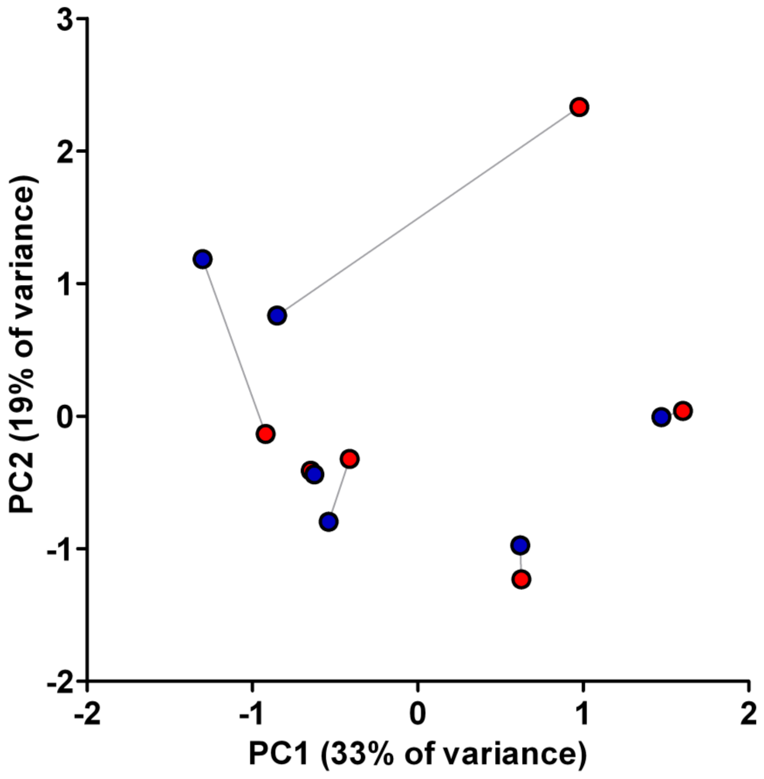

Fig. A two-dimensional ordination by principal component analysis (PCA) of the mycobiomes, as determined through ITS1 sequencing, of apical (N = 6; red) and coronal root segments (N = 6; blue) were not statistically significantly different ($F = 0.327$, $p = 0.966$, PERMANOVA). Principal components PC1, PC2 and PC3 (not shown) explained 33%, 19% and 13% of the overall variance among samples, respectively. Corresponding apical and coronal root segments are connected with a line
